# The impact of optic nerve and related characteristics on disc area measurements derived from different imaging techniques

**DOI:** 10.1371/journal.pone.0190273

**Published:** 2018-01-17

**Authors:** Michael Yapp, George Rennie, Michael P. Hennessy, Michael Kalloniatis, Barbara Zangerl

**Affiliations:** 1 Centre for Eye Health, UNSW Australia, Sydney, New South Wales, Australia; 2 School of Optometry and Vision Science, UNSW Australia, Sydney, New South Wales, Australia; 3 Royal Prince Alfred Hospital, Camperdown, New South Wales, Australia; 4 Department of Ophthalmology, Prince of Wales Hospital, Randwick, New South Wales, Australia; Bascom Palmer Eye Institute, UNITED STATES

## Abstract

**Purpose:**

Optic nerve head (ONH) assessment and its interpretation in healthy patients and those with glaucoma remains a pivotal topic specifically considering rapid advancements in imaging technologies. We undertook a large-scale, mixed cohort, comparative study to assess the correlation of optic disc measurements between different imaging modalities and investigated the impact of patient and disc associated parameters.

**Methods:**

ONH sizes were obtained from one randomly selected eye of each of 209 patients using stereophotography, confocal scanning laser ophthalmoscopy and two different optical coherence tomographers (OCT). Patient related data, glaucoma status and optic disc variables, specifically oblique insertion, torsion, presence of beta PPA and spherical equivalent were recorded. Measurements between imaging modalities were analysed using Pearson correlation, linear regression analysis and Blend-Altman plots. Individual variables were compared applying multivariate regression analysis, ANOVA and chi square statistics was used to determine correlations between patient and clinical characteristics.

**Results:**

Absolute measurements significantly differed between imaging modalities generally producing smaller measurements for OCT derived measurements of Bruch’s membrane opening (BMO). Pairwise correlations between imaging modalities were between 0.83 and 0.93 for discs without myopia, oblique insertion, or beta PPA. These features impacted on measurements for individual modalities and consequently contributed to inconsistencies and variability.

**Conclusion:**

In comparison to planimetry, OCT derived BMO measurements are more variable in the presence of oblique insertion, beta PPA or magnification errors due to myopia. Impact of these factors, however, differs between instruments and needs to be considered to accurately interpret optic disc features in particular within the context of glaucoma diagnosis.

## Introduction

Examination of the optic nerve head (ONH) is a gold standard in the evaluation of aspects of ocular and neurological health, in particular for glaucoma. The measurement of ONH size and orientation is a fundamental component of this assessment due to its role in defining the neuroretinal rim (NRR) and thereby the cup-to-disc (CD) ratio. [1, 2] While absolute measurements are reserved for post-mortem studies, the size of the ONH can be clinically defined by measuring the vertical height with funduscopy. [3] At the same time retinal photography and confocal microscopy (HRT3) allow measurement of the disc area through user delineation of the visible disc margins (planimetry). [4, 5] With the development of Optical Coherence Tomography (OCT) however it has become apparent that the visible disc margin does not necessarily correlate to the opening of the scleral ring as defined by Bruch’s membrane opening (BMO). [6, 7] As a consequence, the optic disc margin is no longer uniformly defined and varies depending on the technology and methodology used to describe it. [7, 8]

The optic nerve head as seen clinically is comprised of retinal nerve fibres, astrocytes, blood vessels and connective tissue. As the nerve fibres pass through the ONH, they distribute around the perimeter of the optic nerve leaving a ‘cup’ in the centre shaping an annulus. While a larger cup therefore indicates a relative sparsity of fibres, this distribution is necessarily dependent on the both the size and orientation of the ONH, [2, 9] as well as the orientation of the incoming fibres. [10] Consequently, the size and orientation of the ONH plays a critical role in evaluating the amount of fibres that comprise the NRR in any particular location, even more so in patients suspected to develop glaucoma. With an increasing use of different imaging modalities in clinical practice, [11] the variation of anatomical landmarks contributing to a clinical determination of the optic disc margins and subsequent source and nature of variations of the NRR are critical, particularly with respect to glaucoma. [1, 5, 12–19] A clinically useful ‘gold standard’ for assessing the NRR has yet to be determined.

In the face of fast evolving and increasingly used technology in clinical practice, we conducted a comprehensive assessment on the relationships of optic disc measurement obtained from the Kowa nonmyd WX3D Retinal Camera (KOWA), Heidelberg Retina Tomograph (HRT) and two OCT devices (Heidelberg Spectralis and Carl Zeiss Cirrus) in a mixed population. HRT analysis may only be used by 40–50% of clinicians for glaucoma assessment, compared to approximately 80% utilisation of OCT imaging, [11] but the required operator dependent delineation of the optic disc margin applied with this image modality provides the means to investigate the robustness of optic disc size measurements in comparison to the more readily obtained disc size from stereoscopic fundus photography. Recent studies specifically demonstrated the benefit of OCT analysis of BMO specific parameters, [20, 21] but also indicated higher interobserver variability in patients with glaucoma. [22] Investigation of a large, mixed cohort (n = 209) allowed us to directly assess correlations in optic disc dimensions between different imaging modalities and investigate the impact of patient and ONH related factors, for instance presence of glaucoma, peri-papillary atrophy (PPA) or optic disc tilt, on such measurements. The outcomes of this study provide insights on the interchangeability of measurements from different devices, and to establish clinical factors affecting the interpretation of the ONH appearance for accurate clinical diagnosis and support integration of adjunct imaging into every day clinical practice.

## Materials and methods

### Patient cohort

Of 359 patients seen at the Centre for Eye Health (CFEH) for ONH examination between September 2014 and January 2015, 209 met the study inclusion criteria consisting of age older than 18, prescription between ±6.00 spherical dioptres, up to 2 dioptres of astigmatism and disc area between 1.0 mm^2^ and 3.5 mm^2^ as measured on either Cirrus or Spectralis OCT based on the limitations in sizes validated for either instrument. Exclusion criteria entailed ONH pathology other than glaucoma for the glaucoma group, or ocular pathology which may affect image quality, e.g. significant cataracts or corneal scarring. Images with movement artefacts through the optic nerve head or of poor quality according to the respective Instrument Quality Scores (Cirrus OCT scans of signal strength less than 6, Spectralis OCT scans of less than 10dB signal to noise ratio and HRT3 ONH scan quality category of ‘poor’ or ‘very poor’) were also excluded.

All patients underwent a complete ONH assessment including family and medical history, visual acuity (VA), intraocular pressure (IOP), autorefraction, autokeratometry, central corneal thickness, gonioscopy, slit lamp and dilated funduscopic assessment. In addition, Standard Automated Perimetry SITA standard 24–2 threshold testing (Carl Zeiss Humphrey Visual Field Analyser, Carl Zeiss Meditec, Dublin, CA), HRT3 (Heidelberg Engineering, Heidelberg, Germany), Spectralis (Heidelberg Engineering, Heidelberg, Germany) and Cirrus OCT (Carl Zeiss Meditec, Dublin, CA) imaging was performed in concordance with the diagnostic glaucoma protocol implemented at CFEH which has been shown to result in a less than 8% overall false positive rate. [23] As previously described, patients were diagnosed with existing glaucoma on the bases of glaucomatous ONH changes and corresponding visual field defects by a highly trained optometrist in consultation with a senior optometrist and consulting ophthalmologist. Patients presenting with changes which were suspicious of but not sufficient for a diagnosis of glaucoma were categorised as glaucoma suspects. All patients included in the study provided written informed consent in accordance with the ethical standards of the Declaration of Helsinki. The study was approved by the Human Research Ethics Advisory at the University of New South Wales (UNSW), Sydney, Australia. Protocol number: 08/2016/36.

### Data collection

For each of the 209 patients included in the study, one eye was chosen at random and laterality was denoted together with the gender and ethnicity of the patient, glaucoma status, refractive error (RE), corneal curvature and the presence or absence of beta PPA. Torsion was measured as a rotation of the disc and considered present if it was beyond 15 degrees in the vertical meridian. Although optic disc tilt was also noted, these discs were further characterised as tilted discs are often defined by different parameters both clinically and in the literature. Optic disc tilt typically comprises two components: oblique insertion and torsion (rotation). [24] Oblique insertion is due to a disparity between the maximum and minimum elevations of the surface of the disc. This is difficult to measure clinically and therefore defined by the surrogate variable ovality, defined as the difference between disc height and width. Oval discs however do not necessarily need to be obliquely inserted. Since the degree of oblique insertion was not a focus of this study, oblique insertion was documented as present or absent from stereoscopic assessment of the disc photos by two independent trained observers. In all cases, equivocal results were reviewed by a third clinician masked to the original classifications.

ONH stereophotography was obtained with the Kowa nonmyd WX3D Retinal Camera (Kowa Optimed Europe Ltd, Sandhurst, Berkshire, UK) and the disc area was manually determined utilizing the corresponding Kowa 3D analysis software. Magnification properties were corrected for by the instruments inherent algorithm incorporating average central corneal curvature and spherical equivalent. HRT3 ONH analysis was performed by manual designation of the optic disc margin through Heidelberg Eye Explorer (Version 1.9.10.0; Heidelberg Engineering, Heidelberg, Germany). Magnification properties on the HRT were corrected for utilising the instruments internal algorithm utilising central average corneal curvature. OCT scans were obtained from the Heidelberg Spectralis OCT (Heidelberg Engineering, Heidelberg, Germany), incorporating retinal tracking technology and delineating the disc margin by taking 24 high resolution radial scans with automated BMO detection acquired through 40,000 A-scans per second. The disc margins were created through circumferentially interpolation between these 48 data points. The Spectralis software algorithm accounts for magnification correction by incorporating the average central corneal curvature as well as the focus setting of the instrument. Images from the Zeiss Cirrus OCT (Carl Zeiss Meditec, Dublin, CA) were acquired through 27,000 A-scans per second, also incorporating retinal tracking. The disc margin was automatically delineating as the edge of Bruch’s membrane from the optic disc cube (200x200) scan protocol. OCT delineation of BMO was not manually adjusted from either instruments software analysis. The Cirrus applies a standard magnification factor of 3.5 degree/mm, but has no inherent algorithm to correct for eye specific magnification properties.

### Statistical analysis

Data analysis was performed using SPSS (Version 23; IBM corporation, Chicago, USA). One-way ANOVA was used to investigate the impact of individual variables on disc size. Multivariate analysis was subsequently performed including all variables with significant results to estimate quantitative effects. Pearson correlation, linear regression analysis, and Bland-Altman were utilised to assess differences in optic disc measurements between imaging modalities. Cases significantly deviating from identified correlations were identified using outlier analysis (>2 standard deviations from the regression line) and manually reviewed. Chi square statistics was employed to investigate correlations between individual patient and clinical characteristics.

## Results

**Fig 1:**
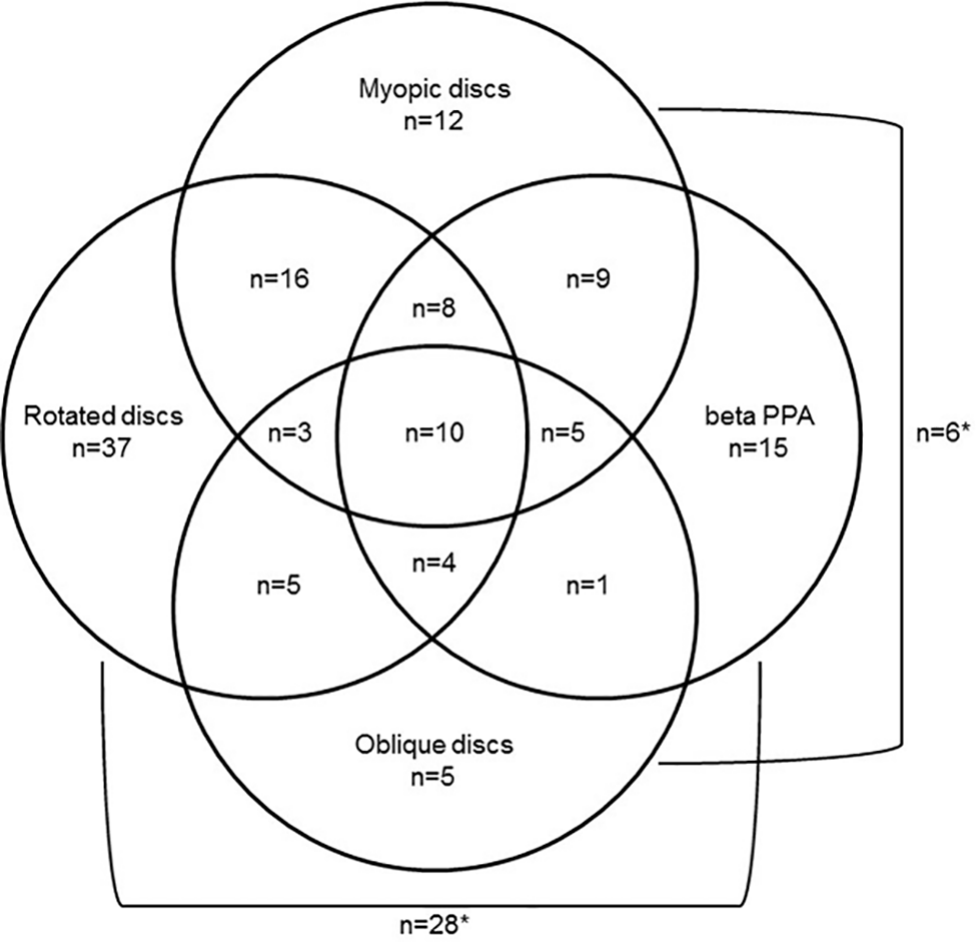
Presence of myopia, tilt, oblique insertion, and beta PPA in examined optic discs. Of all 209 investigated optic discs 69 were myopic, 111 tilted, 39 obliquely inserted and 80 presented with beta PPA, while 45 were not affected by any of these features. Distribution of these characteristics is displayed in a Venn diagram with 69 eyes characterised by a single change, 65 afflicted by two changes, 20 eyes displaying three and 10 eyes presented with all four of the assessed features. The combinations of myopia and oblique insertion as well as tilted discs and beta PPA were found to be significantly associated in pairwise comparison of the complete data set (*).

The mean age of the 209 patients included in this study was 52.8 (±11.2) years with 117 (56%) being male and 135 (65%) identifying as Caucasian, the remainder of Asian ethnicity (Table 1). In total, 20 eyes had glaucomatous changes, 167 eyes were reported at risk of developing glaucoma and 22 eyes did not have noticeable changes. A third of the eyes (n = 69, 33%) was classified as myopic (refractive error ≤ -1) including 23 (11%) of eyes being at least moderately myopic (refractive error ≤ -3). Obliquely inserted disc were more frequently observed with Asian individuals (*Χ*^2^(1) = 9.2, *p* = 0.002), myopic RE (*Χ*^2^(2) = 17.6, *p* = 0.00015) or in eyes with glaucomatous changes (*Χ*^2^(1) = 14.3, *p*<0.0001). Beta PPA was more frequently identified in Glaucomatous and rotated discs (*Χ*^2^(1) = 9.4, *p* = 0.002 and *Χ*^2^(1) = 4.6, *p* = 0.032, respectively). A total of 10 eyes were myopic with rotated, obliquely inserted ONHs with beta PPA, another 45 had none of these features, while the remainder displayed varying combinations of these features (Fig 1).

**Table 1:** Descriptive statistics for 209 patients included in the current study.

	n = 209
**Male gender (%)**	117 (56.0)
**Ethnicity (%)**	
Caucasian	135 (64.6)
Asian	74 (35.4)
**Glaucoma Status (%)**	
Glaucoma	20 (9.6)
Suspect	167 (79.9)
Normal	22 (10.5)
**Right eye (%)**	111 (53.1)
**Beta PPA (%)**	80 (38.3)
**Oblique insertion (%)**	39 (18.7)
**Age, years (SD^a^)**	52.8 (11.2)
**Mean K, mm (SD^a^)**	7.76 (0.25)
**Refractive error, D (SD^a^)**	-0.53 (1.9)
**Optic disc rotation, degree (SD^a^)**	20.8 (21.1)

^a^SD: Standard deviation

**Fig 2:**
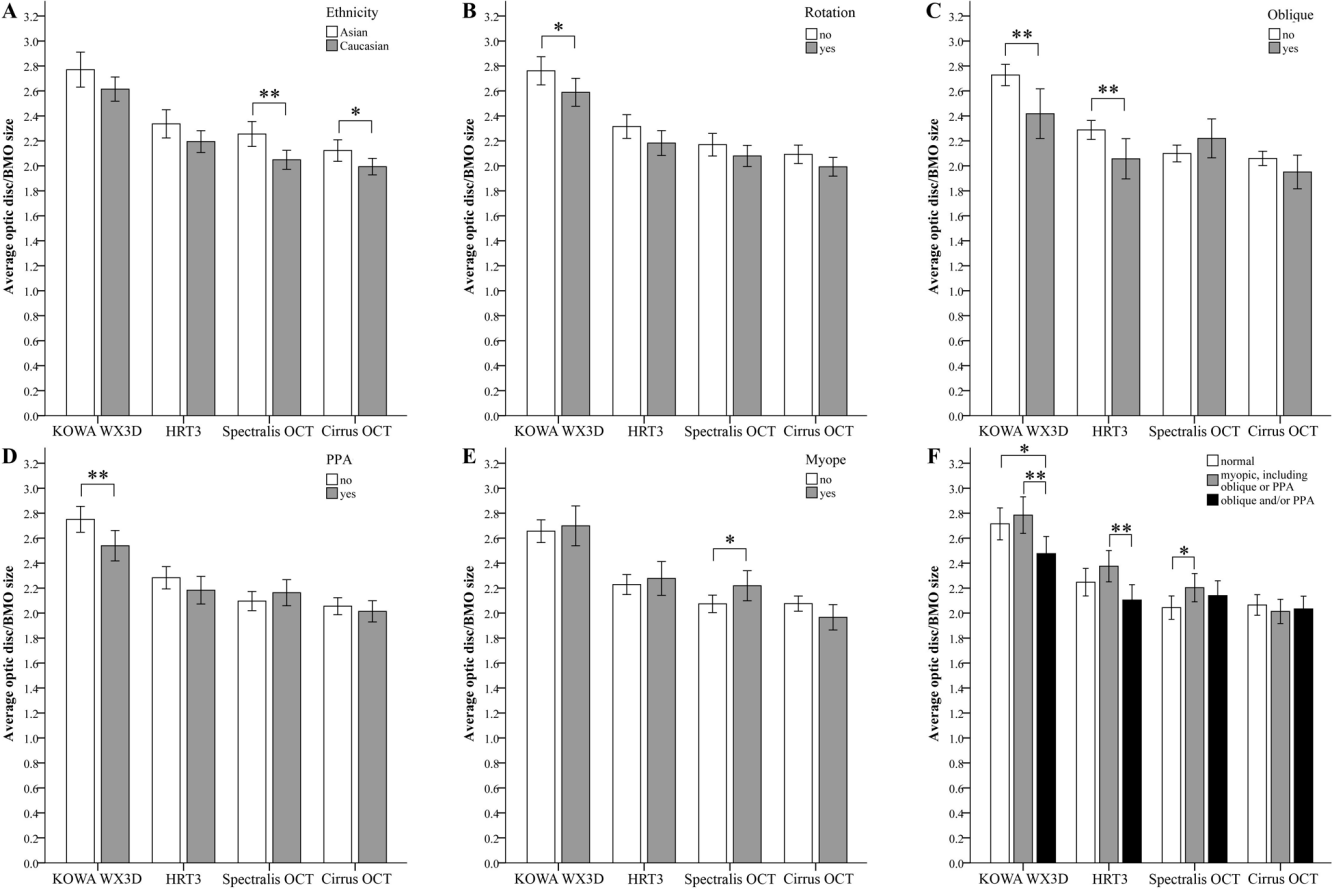
Influence of optic disc characteristics on average disc/BMO sizes measured with four different imaging modalities. Optic disc sizes of 209 participants were measured using KOWA stereoscopic photography and HRT3 topographic imaging and corresponding BMO measurements obtained with the Spectralis and Cirrus OCT. Differences in average size measurements was examined for each imaged modality based on patient ethnicity (**A**), absence or presence of optic disc tilt (**B**), oblique optic disc insertion (**C**), absence or presence of beta PPA (**D**), refraction error of equal or less than -1 defined as myopia (**D**). Of these, myopia as well as oblique insertion and/or presence of beta PPA were associated with significant and ***instrument specific*** differences in average measurement sizes (**E**). **Bars**: 95% confidence interval; **Asterisks**: Significant difference in average measurement size using ANOVA analysis, * = p<0.05, ** = p<0.01.

Measurements of optic discs significantly differed between imaging modalities (paired t-test, *p*<0.0001), resulting on average in larger areas with stereophotography and smaller values for BMO determined by OCT (Table 2). Gender, laterality, or glaucoma status did not individually affect measurement size, but average measurements were larger in patients of Asian ethnicity compared to Caucasian ethnicity with all instruments (Fig 2A), although statistically significant only for BMO sizes (Table 2). Rotated optic discs, oblique insertion, and presence of beta PPA generally were associated with smaller disc/BMO size (Fig 2B–2D), but significant correlation was mainly restricted to measurements from KOWA (Table 2). Multivariate linear regression analysis suggests that ethnicity and oblique insertion are the main variables impacting optic disc measurements for instruments dependent on observer delineation, while ethnicity and myopia impact BMO sizes (Table 2). Additionally, optic disc rotation is correlated with BMO measurements with the Cirrus OCT (Table 2). Two main differences were noted comparing measurements between instruments: (1) oblique insertion and beta PPA displayed a trend towards larger BMO measurements for data obtained from Spectralis OCT (Fig 2C and 2D) and (2) myopic RE was associated with larger measurements, for all but BMO calculated with Cirrus OCT (Fig 2E), albeit significant for Spectralis OCT only (Table 2). These deviations are likely based on differences in the effect of specific optic disc features, foremost myopia and the presence of oblique insertion or beta PPA, on the average disc size/BMO measurements between imaging modalities (Fig 2F).

**Table 2:** Average optic disc area, standard deviation (SD) and 95% confidence interval (CI) for 209 optic discs measured with different modalities.

	KOWA WX3D	HRT3	Spectralis OCT	Cirrus OCT
**Disc area**				
Mean mm^2^ (SD)	2.67 (0.58)	2.24 (0.51)	2.12 (0.45)	2.04 (0.39)
95% CI	2.59–2.75	2.18–2.31	2.06–2.18	1.99–2.09
**ANOVA p-value**				
Gender	0.151	0.126	0.511	0.090
Eye laterality	0.106	0.686	0.801	0.651
Glaucoma status	0.349	0.778	0.797	0.303
Ethnicity	0.054	0.053	**0.001**	**0.021**
Rotation	**0.044**	0.059	0.152	0.063
Oblique	**0.003**	**0.010**	0.132	0.115
Beta PPA	**0.014**	0.168	0.295	0.457
Myopia	0.333	0.727	**0.024**	0.129
**Multivariate regression analysis, coefficient (p-value)**				
Gender	-0.111 (0.159)	-0.101 (0.149)	-0.044 (0.477)	-0.095 (0.074)
Eye laterality	-0.136 (0.078)	-0.310 (0.654)	0.042 (0.486)	-0.028 (0.597)
Glaucoma status	-0.033 (0.711)	0.010 (0.896)	-0.082 (0.246)	-0.077 (0.209)
Ethnicity	**0.205 (0.013)**	**0.187 (0.011)**	**0.205 (0.002)**	**0.159 (0.005)**
Rotation	-0.141 (0.073)	-0.126 (0.071)	-0.121 (0.052)	**-0.115 (0.030)**
Oblique	**-0.366 (0.001)**	**-0.290 (0.003)**	0.042 (0.620)	-0.078 (0.289)
Beta PPA	-0.148 (0.076)	-0.055 (-0.744)	0.101 (0.124)	0.009 (0.877)
Myopia	0.106 (0.070)	0.072 (0.165)	**0.101 (0.029)**	**-0.078 (0.049)**

Impact of assessed clinical features on recorded optic disc or BMO sizes respectively was investigated for each modality applying ANOVA analysis using multivariate linear regression for optic disc features in addition to gender, ethnicity, eye laterality, and glaucoma status. The resulting coefficients of variation and *p-values* are indicated for each optic disc feature with significant values bolded.

**Fig 3:**
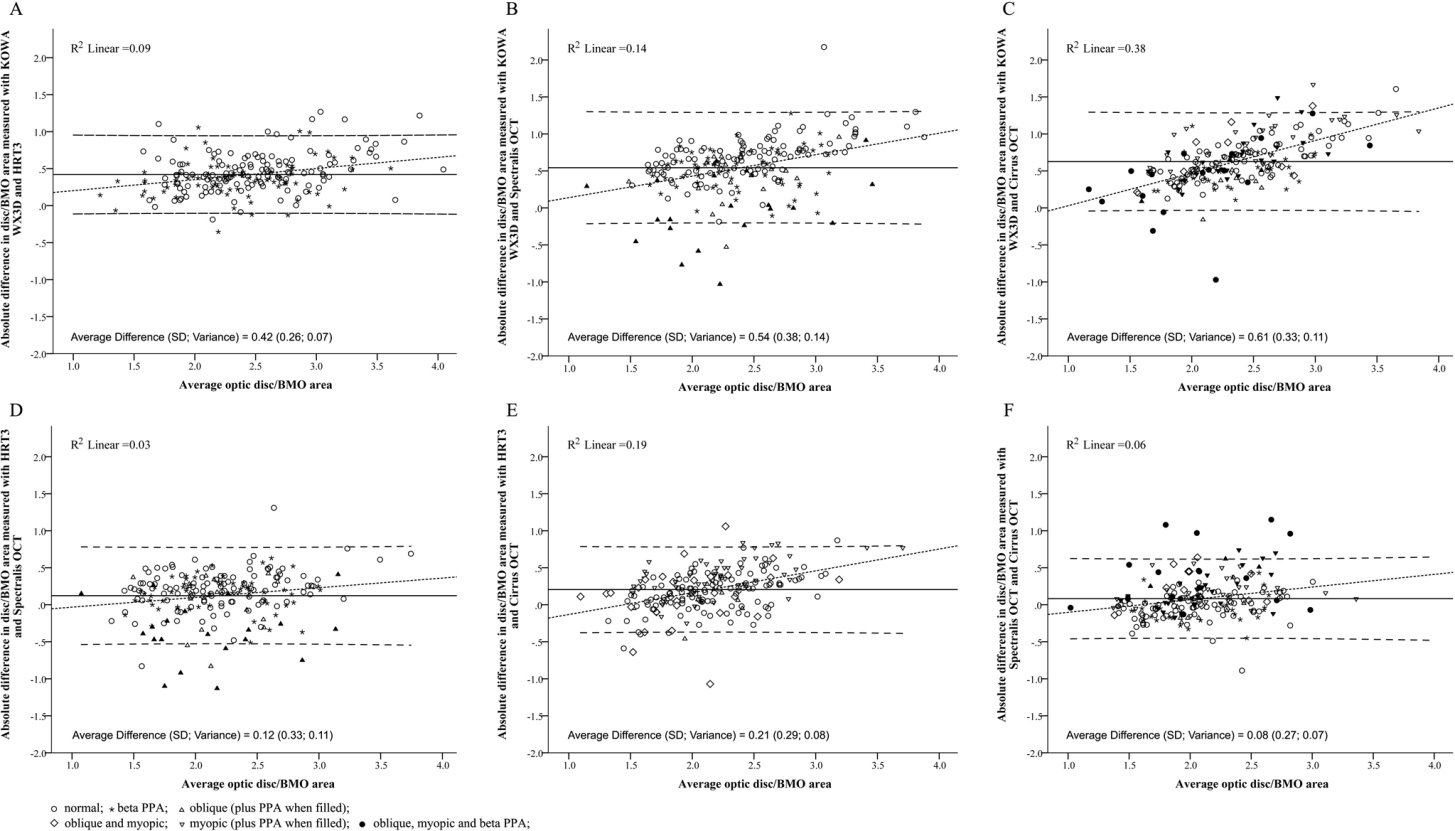
Pairwise comparison of optic disc/BMO measurements between investigated imaging modalities. Bland Altman analysis was applied to examine differences in optic disc/BMO measurements between each pair of imaging devices by plotting the absolute difference in measurements over the average measurement (both mm^2^). The largest differences independent of absolute measurement size were observed with comparisons of either HRT3 (**A**), Spectralis OCT (**B**), or Cirrus OCT (**C**) to those derived from theKOWA. Absolute measurements are closer between HRT3 and either Spectralis OCT (**E**) or Cirrus OCT (**F**), while BMO measurements obtained from the two OCT instruments (**G**) resulted in the smallest absolute difference with size measurements as well as narrowest confidence interval. For each comparison, the average difference (solid horizontal line) and 95% confidence interval (dashed horizontal lines). Displayed regression lines (dashed line) and associated regression coefficient (R^2^) are indicative of a potential bias in comparative measurements with absolute size, which is most prominent in comparisons with Cirrus OCT BMO measurements (C, E, F). Additionally, optic disc features determined to significantly influence the average difference in size measurements using ethnicity, myopia, optic disc tilt, oblique insertion, and presence of beta PPA as independent variables in a multivariate regression analysis.

Correlation of size measurements obtained from different image modalities was weakest between Spectralis OCT BMO sizes to other instruments, while all pairwise correlations showed similar strength if calculated from 82 optic discs that were not diagnosed with myopia, oblique insertion, or beta PPA (Table 3A). Comparisons involving measurements from the KOWA resulted in higher absolute differences than those between other instruments (Fig 3, intersection of solid horizontal lines). Variability of measurement differences was lowest and equal (variance = 0.07) for comparisons either between operated dependent lineation of optic disc sizes measured with KOWA and HRT3 (Fig 3A), or between automatically determined BMO sizes obtained from Spectralis and Cirrus OCT (Fig 3F). Multivariate linear regression analysis considering ethnicity, rotation, oblique insertion, beta PPA and myopia was subsequently applied to identify parameters that significantly influenced the measurement difference for each pairwise comparison highlighting oblique insertion, myopia, and presence of beta PPA as modifying parameters for individual associations after correcting for other effects (Fig 3, Table 4). While ethnicity was significantly associated with all optic disc size measurements (Table 2), it impacted each measurement method similarly (Fig 2), therefore not affecting comparisons between image modalities (Table 4). Both correlation analysis and multivariate regression analysis suggest that comparison of optic disc measurement between KOWA and HRT3 was least influenced by investigated disc features (Table 3B, Fig 3).

**Table 3:** Pairwise correlation of optic disc or BMO area measurements respectively from different modalities and impact of optic disc features.

**A** Correlation measured by PCC for all discs (n = 209) and those without myopia, oblique insertion, or beta PPA (normal, n = 82)					
	normal	**KOWA WX3D**	**HRT3**	**Spectralis OCT**	**Cirrus OCT**
all discs					
**KOWA WX3D**			0.90	0.88	0.93
**HRT3**		0.89		0.83	0.87
**Spectralis OCT**		0.76	0.77		0.90
**Cirrus OCT**		0.84	0.82	0.80	
**B** Changes to PCC with oblique disc insertion (n = 39) and beta PPA (n = 80)					
	Oblique	**KOWA WX3D**	**HRT3**	**Spectralis OCT**	**Cirrus OCT**
beta PPA					
**KOWA WX3D**			0.93	0.65	0.72
**HRT3**		0.90		0.61	0.70
**Spectralis OCT**		0.69	0.72		0.68
**Cirrus OCT**		0.78	0.78	0.72	

All correlations were statistically significant (p<0.001)

**Table 4:** Optic disc variables impacting on differences in optic disc size measurements between different imaging modalities.

CoV	KOWA WX3D	HRT3	Spectralis OCT	Cirrus OCT
*p-value*				
**KOWA WX3D**				
Ethnicity		0.022	0.002	0.047
Rotation		-0.015	-0.030	-0.034
Oblique		-0.081	**-0.395**	**-0.272**
Beta PPA		**-0.096**	**-0.228**	**-0.144**
Myopia		0.039	0.013	0.188
**HRT3**				
Ethnicity	*0*.*576*		-0.019	0.025
Rotation	*0*.*691*		-0.016	-0.020
Oblique	*0*.*102*		**-0.314**	**-0.190**
Beta PPA	***0*.*013***		**-0.132**	-0.048
Myopia	*0*.*158*		**-0.025**	**0.149**
***Spectralis OCT***				
Ethnicity	*0*.*960*	*0*.*660*		0.045
Rotation	*0*.*505*	*0*.*704*		-0.004
Oblique	***<0*.*001***	***<0*.*001***		**0.123**
Beta PPA	***<0*.*001***	***0*.*003***		**0.083**
Myopia	*0*.*693*	*0*.*414*		**0.174**
***Cirrus OCT***				
Ethnicity	*0*.*283*	*0*.*533*	*0*.*186*	
Rotation	*0*.*406*	*0*.*607*	*0*.*903*	
Oblique	***<0*.*001***	***<0*.*001***	***0*.*004***	
Beta PPA	***0*.*001***	*0*.*225*	***0*.*012***	
Myopia	***<0*.*001***	***<0*.*001***	***<0*.*001***	

Multivariate linear regression analysis was performed to estimate the quantitative effect of variables significantly impacting optic disc measurements of individual imaging modalities in univariant analysis (Table 2, Fig 2) on the correlation of measurements between image modalities. Resulting coefficients of variation (CoV) and corresponding p-values are indicated for each pairwise comparison of measurements.

**Fig 4:**
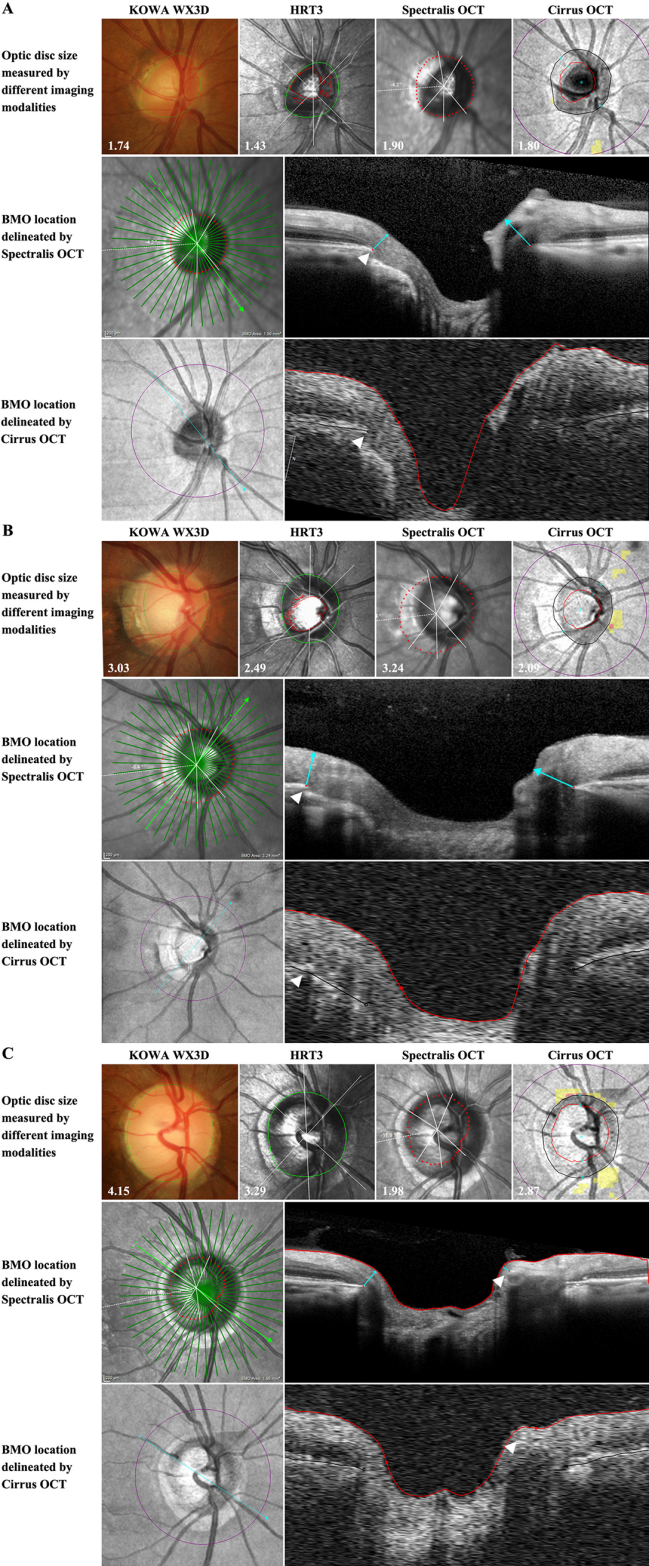
Representative examples of optic discs leading to significant difference in size interpretation with different imaging modalities. **(A)** Comparative images of the KOWA, HRT, Spectralis and Cirrus highlighting the larger disc sizes on the OCTs in comparison to the KOWA and HRT. The discrepancy is located supero-temporally and related to oblique insertion resulting in the location of BMO (white arrows) not correlating to the location of the visible disc margin. (**B)** Comparative images of the KOWA, HRT, Spectralis and Cirrus highlighting the difference in the two OCTs automated localisation of BMO. The higher resolution Spectralis line scan enables automated detection closer to a trained observer’s manual assessment (white arrows). (**C)** Comparative images of the KOWA, HRT, Spectralis and Cirrus highlighting an outlier in the Spectralis measurement of disc size due to incorrect delineation of BMO (white arrow) related to the presence of overlying vasculature.

A total of 16 optic discs obtained a comparative value outside the 99 percentiles for any of the pairwise comparisons consisting of 3 normal, 1 oblique, 1 myopic, 2 oblique and myopic, and 3 myopic optic discs with PPA present; the remaining 6 optic discs were myopic, obliquely inserted, with beta PPA. Optic disc measurements were almost always larger than BMO sizes, with the most noticeable deviations occurring when this was reversed and the BMO size was larger than the optic disc size (Fig 3B–3E, below lower dashed line). The same obliquely inserted optic discs were commonly identified with analysis also showing an association with the presence of beta PPA for the Spectralis OCT, and myopia for the Cirrus OCT (Fig 4A). The variation in size measurements was commonly due to a temporal displacement of BMO in comparison to the visible disc margin. There were also however a number of discs measured as markedly larger with the Spectralis in comparison to the Cirrus (Fig 3F). While also associated with oblique insertion and beta PPA, this discrepancy was as a result of notable variations between the instruments inherent delineation of BMO (Fig 4B). The Spectralis closer resembled trained observer manual segmentation in these cases likely due to the higher resolution obtained with the scan pattern utilised by the respective instruments. As previously stated, the comparison between KOWA and HRT3 comparison was least affected by optic disc feature resulting in a smaller margin of error without clear outliers (Fig 3A). Where discrepancies existed, the majority of discs were associated with high myopia combined with oblique insertion or rotation. Another simple disc was flagged in comparisons with the Spectralis OCT (Fig 3B, 3D and 3F; arrow head) due to an error with BMO delineation related to an anatomical vessel variation (Fig 4C).

## Discussion

This comprehensive study demonstrated that optic disc size measurements obtained in a large, mixed cohort are well correlated between image modalities based on either operator dependent optic disc margin delineation or BMO with mean differences across all comparisons of up to 0.6 mm^2^. While operator-dependent disc sizes obtained from stereoscopic photography and HRT resulted in better correlation of the measurements (Table 3), BMO sizes calculated from the two investigated OCT instruments exhibited smaller absolute measurement differences (Fig 3). OCTs have been shown to produce relatively smaller disc area measurements both in normal and glaucoma patients, [1, 25] especially for larger discs. [18, 26] Our results however identified a notable number of exceptions, where eyes with obliquely inserted discs in conjunction with beta PPA and myopia, had OCT measurements that exceeded those obtained from disc photography or HRT (Fig 3A). This suggests that the more commonly temporally external oblique configuration of the border tissue of Elschnig may lead to a larger measurement relative to. [8, 27]

Oblique optic disc insertion has also been suggested to contribute to smaller optic disc parameters on OCT measurements in glaucoma patients. [28] Our results support this finding for measurements obtained from the Cirrus OCT, but not for Spectralis OCT (Fig 2C). Analysis of the two OCT data sets showed that BMO sizes obtained from Cirrus and Spectralis OCT were strongly correlated, yet still significantly different (*p*<0.0001) with the latter on average providing marginally larger measurements. Previous studies have highlighted that these differences are likely to be due to both the instruments internal algorithms as well as scan patterns. [29, 30] Spectralis derived optic disc measurements consistently produced the weakest correlations and the greatest number of outliers with beta PPA being a main contributing factor. Analysis of these outliers showed that variations in disc area measurements between the two instruments resulted from markedly different delineation of the BMO location in the areas of beta PPA (Fig 3B). This difference could be a consequence of the higher resolution images on the Spectralis, a result of the variation in methods of data acquisition allowing for a more accurate instrument based delineation of BMO. Consequently, the Spectralis data set is more likely to match manual OCT delineation, [31] and accounts for the larger comparative area measurements in obliquely inserted discs due to its association with temporal beta PPA. This is consistent with previous studies on temporal external oblique configurations. [8, 27] Critically, it may also be a key factor in studies showing correlations between myopia, disc tilt and RFNL thickness measurements in particular in the temporal sector, [10, 32] as well as an increased specificity of the Spectralis BMO assessment. [21]

Interestingly, our results suggest that myopic eyes result in a smaller average disc size on the Cirrus, contrary to the trend observed with the other instruments studied. Although none of the instruments inbuilt algorithms utilise axial length, the KOWA, Spectralis and HRT utilise inbuilt magnification correction for keratometry values. The mean K values of the myopic group however was not significantly different in comparison and therefore is unlikely to account for the variation. The Spectralis and KOWA instruments also correct magnification using spherical equivalent refraction, with the HRT’s method also approximating Bennet’s method, which results in an increase in disc size in myopic eyes when compared to uncorrected values. [33] It is therefore possible that this difference in myopic discs on average measuring smaller on the Cirrus is based on a lack of an inherent magnification correction. Markedly, our results showed no significant difference between the disc size measurements in the healthy, glaucoma suspect and glaucomatous subgroups on all four investigated instruments. The glaucomatous eyes were more frequently had associated beta PPA in this cohort. Glaucomatous eyes are also frequently associated with myopia and more prone to peri-papillary atrophy. [34, 35] As noted in the earlier discussion, the Spectralis OCT suggested eyes with oblique insertion and beta PPA are more likely to exhibit a disparity between the visible disc margins and BMO. This suggests that in eyes with oblique insertion and beta PPA, particular care should be taken in assessing the NRR for glaucoma as it is more likely that the visible disc margins do not match BMO in these discs.

## Conclusion

The current study confirmed that optic disc measurements using planimetry and OCT derived BMO generally show good correlation. As with other studies, OCT tends to produce relatively smaller disc area measurements. The presence of oblique insertion and beta PPA was linked to notable variation between BMO and the clinically visible disc margin on the Spectralis. This difference was less evident on the Cirrus, possibly because it has a lower resolution which affects the accuracy of the automatic delineation of BMO. However, disc sizes obtained from the Cirrus OCT were more likely to be affected by magnification errors in eyes with myopia.

The clinical ramifications of these differences, if any, are yet to be fully determined. Clinicians should be aware of these variations when assessing the ONH, in particular with regards to assessing: the integrity of the NRR; OCT automated segmentation; and normative analysis specifically when glaucoma is being identified in eyes with oblique inserted discs with PPA.

## Supporting information

S1 Anonymized data set Original data used for analysis in this manuscript. (PDF)

## References

[1] Hoffmann EM, Zangwill LM, Crowston JG, Weinreb RN. Optic disk size and glaucoma. Survey of ophthalmology. 2007;52(1):32–49. doi: 10.1016/j.survophthal.2006.10.002 17212989 10.1016/j.survophthal.2006.10.002 PMC1850981

[2] Jonas JB, Budde WM. Diagnosis and pathogenesis of glaucomatous optic neuropathy: morphological aspects. Prog Retin Eye Res. 2000;19(1):1–40. 10614679 10.1016/s1350-9462(99)00002-6

[3] Ansari-Shahrezaei S, Maar N, Biowski R, Stur M. Biomicroscopic measurement of the optic disc with a high-power positive lens. Invest Ophthalmol Vis Sci. 2001;42(1):153–7. 11133860

[4] Bartling H, Wanger P, Martin L. Measurement of optic disc parameters on digital fundus photographs: algorithm development and evaluation. Acta ophthalmologica. 2008;86(8):837–41. doi: 10.1111/j.1755-3768.2007.01146.x 19086927 10.1111/j.1755-3768.2007.01146.x

[5] Jonas JB, Mardin CY, Grundler AE. Comparison of measurements of neuroretinal rim area between confocal laser scanning tomography and planimetry of photographs. Br J Ophthalmol. 1998;82(4):362–6. 9640181 10.1136/bjo.82.4.362 PMC1722555

[6] Hu Z, Abramoff MD, Kwon YH, Lee K, Garvin MK. Automated segmentation of neural canal opening and optic cup in 3D spectral optical coherence tomography volumes of the optic nerve head. Invest Ophthalmol Vis Sci. 2010;51(11):5708–17. doi: 10.1167/iovs.09-4838 20554616 10.1167/iovs.09-4838 PMC3061507

[7] Strouthidis NG, Yang H, Reynaud JF, Grimm JL, Gardiner SK, Fortune B, et al Comparison of clinical and spectral domain optical coherence tomography optic disc margin anatomy. Invest Ophthalmol Vis Sci. 2009;50(10):4709–18. doi: 10.1167/iovs.09-3586 19443718 10.1167/iovs.09-3586 PMC2751811

[8] Reis AS, Sharpe GP, Yang H, Nicolela MT, Burgoyne CF, Chauhan BC. Optic disc margin anatomy in patients with glaucoma and normal controls with spectral domain optical coherence tomography. Ophthalmology. 2012;119(4):738–47. doi: 10.1016/j.ophtha.2011.09.054 22222150 10.1016/j.ophtha.2011.09.054 PMC3319857

[9] Jonas JB, Gusek GC, Naumann GO. Optic disc, cup and neuroretinal rim size, configuration and correlations in normal eyes. Invest Ophthalmol Vis Sci. 1988;29(7):1151–8. 3417404

[10] Hwang YH, Yoo C, Kim YY. Myopic optic disc tilt and the characteristics of peripapillary retinal nerve fiber layer thickness measured by spectral-domain optical coherence tomography. J Glaucoma. 2012;21(4):260–5. doi: 10.1097/IJG.0b013e31820719e1 21623226 10.1097/IJG.0b013e31820719e1

[11] Jamous KF, Kalloniatis M, Hayen A, Mitchell P, Stapleton FJ, Zangerl B. Application of clinical techniques relevant for glaucoma assessment by optometrists: concordance with guidelines. Ophthalmic Physiol Opt. 2014;34(5):580–91. doi: 10.1111/opo.12146 25103462 10.1111/opo.12146

[12] Barkana Y, Harizman N, Gerber Y, Liebmann JM, Ritch R. Measurements of optic disk size with HRT II, Stratus OCT, and funduscopy are not interchangeable. Am J Ophthalmol. 2006;142(3):375–80. doi: 10.1016/j.ajo.2006.03.065 16935579 10.1016/j.ajo.2006.03.065

[13] Leung CK, Cheng AC, Chong KK, Leung KS, Mohamed S, Lau CS, et al Optic disc measurements in myopia with optical coherence tomography and confocal scanning laser ophthalmoscopy. Invest Ophthalmol Vis Sci. 2007;48(7):3178–83. doi: 10.1167/iovs.06-1315 17591887 10.1167/iovs.06-1315

[14] Moghimi S, Hosseini H, Riddle J, Lee GY, Bitrian E, Giaconi J, et al Measurement of optic disc size and rim area with spectral-domain OCT and scanning laser ophthalmoscopy. Invest Ophthalmol Vis Sci. 2012;53(8):4519–30. doi: 10.1167/iovs.11-8362 22577077 10.1167/iovs.11-8362

[15] Neubauer AS, Krieglstein TR, Chryssafis C, Thiel M, Kampik A. Comparison of optical coherence tomography and fundus photography for measuring the optic disc size. Ophthalmic Physiol Opt. 2006;26(1):13–8. doi: 10.1111/j.1475-1313.2005.00339.x 16390477 10.1111/j.1475-1313.2005.00339.x

[16] Resch H, Deak G, Pereira I, Vass C. Comparison of optic disc parameters using spectral domain cirrus high-definition optical coherence tomography and confocal scanning laser ophthalmoscopy in normal eyes. Acta ophthalmologica. 2012;90(3):e225–9. doi: 10.1111/j.1755-3768.2012.02385.x 22458635 10.1111/j.1755-3768.2012.02385.x

[17] Schuman JS, Wollstein G, Farra T, Hertzmark E, Aydin A, Fujimoto JG, et al Comparison of optic nerve head measurements obtained by optical coherence tomography and confocal scanning laser ophthalmoscopy. Am J Ophthalmol. 2003;135(4):504–12. 12654368 10.1016/s0002-9394(02)02093-7

[18] Sharma A, Oakley JD, Schiffman JC, Budenz DL, Anderson DR. Comparison of automated analysis of Cirrus HD OCT spectral-domain optical coherence tomography with stereo photographs of the optic disc. Ophthalmology. 2011;118(7):1348–57. doi: 10.1016/j.ophtha.2010.12.008 21397334 10.1016/j.ophtha.2010.12.008 PMC3129482

[19] Vihanninjoki K, Tuulonen A, Burk RO, Airaksinen PJ. Comparison of optic disc measurements by Heidelberg Retina Tomograph and manual planimetric techniques. Acta Ophthalmol Scand. 1997;75(5):512–5. 9469546 10.1111/j.1600-0420.1997.tb00139.x

[20] Gmeiner JM, Schrems WA, Mardin CY, Laemmer R, Kruse FE, Schrems-Hoesl LM. Comparison of Bruch's Membrane Opening Minimum Rim Width and Peripapillary Retinal Nerve Fiber Layer Thickness in Early Glaucoma Assessment. Invest Ophthalmol Vis Sci. 2016;57(9):OCT575–84. doi: 10.1167/iovs.15-18906 27547890 10.1167/iovs.15-18906

[21] Rebolleda G, Casado A, Oblanca N, Munoz-Negrete FJ. The new Bruch's membrane opening—minimum rim width classification improves optical coherence tomography specificity in tilted discs. Clin Ophthalmol. 2016;10:2417–25. doi: 10.2147/OPTH.S120237 27980390 10.2147/OPTH.S120237 PMC5147415

[22] Reis ASC, Zangalli CES, Abe RY, Silva AL, Vianna JR, Vasconcellos JPC, et al Intra- and interobserver reproducibility of Bruch's membrane opening minimum rim width measurements with spectral domain optical coherence tomography. Acta ophthalmologica. 2017. 10.1111/aos.13464 28650590

[23] Jamous KF, Kalloniatis M, Hennessy MP, Agar A, Hayen A, Zangerl B. Clinical model assisting with the collaborative care of glaucoma patients and suspects. Clin Experiment Ophthalmol. 2015;43(4):308–19. doi: 10.1111/ceo.12466 25362898 10.1111/ceo.12466

[24] Witmer MT, Margo CE, Drucker M. Tilted optic disks. Survey of ophthalmology. 2010;55(5):403–28. doi: 10.1016/j.survophthal.2010.01.002 20621322 10.1016/j.survophthal.2010.01.002

[25] Samarawickrama C, Pai A, Huynh SC, Burlutsky G, Jonas JB, Mitchell P. Measurement of optic nerve head parameters: comparison of optical coherence tomography with digital planimetry. J Glaucoma. 2009;18(8):571–5. doi: 10.1097/IJG.0b013e3181996da6 19826383 10.1097/IJG.0b013e3181996da6

[26] Lee M, Yoo H, Ahn J. Comparison of disc analysis algorithms provided by cirrus OCT and stereo optic-disc photography in normal and open angle glaucoma patients. Curr Eye Res. 2013;38(5):605–13. doi: 10.3109/02713683.2013.769059 23448436 10.3109/02713683.2013.769059

[27] Young M, Lee S, Rateb M, Beg MF, Sarunic MV, Mackenzie PJ. Comparison of the clinical disc margin seen in stereo disc photographs with neural canal opening seen in optical coherence tomography images. J Glaucoma. 2014;23(6):360–7. doi: 10.1097/IJG.0b013e31829484a4 25075462 10.1097/IJG.0b013e31829484a4

[28] Shin HY, Park HY, Park CK. The effect of myopic optic disc tilt on measurement of spectral-domain optical coherence tomography parameters. Br J Ophthalmol. 2015;99(1):69–74. doi: 10.1136/bjophthalmol-2014-305259 25091955 10.1136/bjophthalmol-2014-305259

[29] Agrawal A, Baxi J, Calhoun W, Chen CL, Ishikawa H, Schuman JS, et al Optic Nerve Head Measurements With Optical Coherence Tomography: A Phantom-Based Study Reveals Differences Among Clinical Devices. Invest Ophthalmol Vis Sci. 2016;57(9):OCT413–20. doi: 10.1167/iovs.15-18738 27409500 10.1167/iovs.15-18738 PMC4968925

[30] Savini G, Barboni P, Carbonelli M, Sbreglia A, Deluigi G, Parisi V. Comparison of optic nerve head parameter measurements obtained by time-domain and spectral-domain optical coherence tomography. J Glaucoma. 2013;22(5):384–9. doi: 10.1097/IJG.0b013e31824c9423 22366702 10.1097/IJG.0b013e31824c9423

[31] Garas A, Vargha P, Hollo G. Automatic, operator-adjusted, and manual disc-definition for optic nerve head and retinal nerve fiber layer measurements with the RTVue-100 optical coherence tomograph. J Glaucoma. 2011;20(2):80–6. doi: 10.1097/IJG.0b013e3181d787fd 20436363 10.1097/IJG.0b013e3181d787fd

[32] Law SK, Tamboli DA, Giaconi J, Caprioli J. Characterization of retinal nerve fiber layer in nonglaucomatous eyes with tilted discs. Arch Ophthalmol. 2010;128(1):141–2. doi: 10.1001/archophthalmol.2009.340 20065236 10.1001/archophthalmol.2009.340

[33] Garway-Heath DF, Rudnicka AR, Lowe T, Foster PJ, Fitzke FW, Hitchings RA. Measurement of optic disc size: equivalence of methods to correct for ocular magnification. Br J Ophthalmol. 1998;82(6):643–9. 9797665 10.1136/bjo.82.6.643 PMC1722616

[34] Jonas JB, Naumann GO. Parapapillary retinal vessel diameter in normal and glaucoma eyes. II. Correlations. Invest Ophthalmol Vis Sci. 1989;30(7):1604–11. 2745001

[35] Jonas JB, Nguyen XN, Naumann GO. Parapapillary retinal vessel diameter in normal and glaucoma eyes. I. Morphometric data. Invest Ophthalmol Vis Sci. 1989;30(7):1599–603. 2745000

