# Supplementary material for: The impact of optic nerve and related characteristics on disc area measurements derived from different imaging techniques
Source: PLoS One. 2018 Jan 17;13(1):e0190273. doi: 10.1371/journal.pone.0190273 (PMC5771575; doi:10.1371/journal.pone.0190273)
Supplement: S1 Anonymized data set — (PDF) [file pone.0190273.s001.pdf]

Patient	Age	Eye	Gender	Ethnicity	Disease	Refrative Error	Corneal Curvature (Mean K)	beta PPA	Optic disc rotation (°)	Oblique insertion	KOWA disc size	HRT disc size	Spectralis BMO size	Cirrus BMO size
1	47.9	OD	Male	Asian	Glaucoma suspect	-0.63	8.18	absent	9	absent	4.15	3.29	1.98	2.87
2	68.4	OD	Male	Caucasian	Glaucoma suspect	3.38	7.78	absent	2	absent	2.85	2.19	1.94	2.43
3	56.1	OD	Female	Caucasian	Glaucoma suspect	4	7.8	present	0	absent	3.05	2.54	2.24	2.69
4	69.8	OD	Female	Caucasian	Glaucoma suspect	3.63	7.59	absent	30	absent	2.12	1.52	1.33	1.72
5	56.1	OS	Male	Caucasian	Glaucoma suspect	0.5	7.24	present	19	absent	2.44	2.04	1.82	2.15
6	41.5	OD	Female	Caucasian	Glaucoma suspect	1.13	7.67	absent	6	absent	2	1.7	1.41	1.71
7	53	OS	Male	Asian	Glaucoma suspect	0.25	7.42	present	23	absent	2.31	2.02	1.78	2.06
8	49.4	OD	Male	Asian	Glaucoma suspect	0.5	7.74	absent	24	absent	3.91	3.07	2.68	2.96
9	65.7	OS	Male	Caucasian	Glaucoma suspect	1.63	7.11	absent	0	absent	2.27	1.38	1.48	1.75
10	54.1	OD	Male	Caucasian	Glaucoma suspect	-0.25	7.52	absent	5	absent	2.3	2	1.51	1.78
11	32.7	OD	Male	Caucasian	Normal	-0.13	7.15	absent	74	absent	2.61	2.33	1.84	2.11
12	57.7	OD	Male	Caucasian	Glaucoma	-0.13	7.58	absent	2	absent	2.19	1.77	1.49	1.74
13	56.9	OS	Male	Caucasian	Glaucoma suspect	1.5	7.83	absent	45	present	2.42	2.09	1.81	2.05
14	47.8	OS	Male	Asian	Glaucoma suspect	1.25	7.61	absent	12	absent	2.69	2.43	2.04	2.28
15	51	OD	Male	Caucasian	Glaucoma suspect	1.75	7.1	present	16	absent	2.45	1.92	1.98	2.19
16	68.6	OS	Male	Caucasian	Glaucoma suspect	2.38	7.75	absent	0	absent	2.05	1.65	1.5	1.71
17	65.7	OS	Female	Caucasian	Glaucoma suspect	0.63	7.52	present	16	absent	2.1	1.66	1.51	1.72
18	55.1	OD	Female	Caucasian	Normal	0.5	7.88	present	4	absent	2.37	2	1.62	1.83
19	60.8	OD	Female	Caucasian	Glaucoma suspect	2.5	7.64	present	0	absent	3.06	2.55	2.31	2.51
20	42.7	OS	Male	Caucasian	Glaucoma suspect	-0.63	7.87	absent	38	absent	2.06	1.64	1.45	1.64
21	47.7	OS	Female	Asian	Normal	1.75	7.74	absent	15	present	2.56	2.32	1.98	2.16
22	67	OD	Male	Caucasian	Glaucoma suspect	0.25	7.94	absent	9	absent	2.46	2.09	1.69	1.87
23	75.1	OD	Male	Caucasian	Glaucoma	2	7.69	present	18	absent	2.32	2.05	1.77	1.95
24	55.2	OS	Male	Caucasian	Glaucoma	0.75	7.92	absent	0	present	2.58	2.28	1.89	2.07
25	44.5	OD	Male	Asian	Glaucoma suspect	0.25	7.44	absent	39	present	2.52	2.08	1.83	2.01
26	57.3	OD	Female	Asian	Glaucoma suspect	0	7.52	present	13	absent	3.34	2.64	2.47	2.64
27	58.2	OD	Female	Caucasian	Glaucoma	1.38	7.86	present	14	absent	2.54	2.1	1.75	1.92
28	63.9	OD	Female	Caucasian	Glaucoma suspect	-1.25	7.44	present	0	absent	2.46	2.24	1.79	1.96
29	69.2	OD	Male	Caucasian	Glaucoma suspect	1.63	7.8	present	90	absent	2.22	1.66	1.6	1.76
30	56.6	OS	Female	Caucasian	Glaucoma suspect	1.75	7.75	absent	45	absent	2.34	2.07	1.56	1.72
31	51.3	OS	Male	Caucasian	Glaucoma suspect	0.5	7.35	absent	0	absent	2.87	2.32	2.28	2.43
32	63.6	OS	Female	Caucasian	Glaucoma suspect	-1.38	7.41	absent	20	absent	2.56	1.83	1.93	2.08
33	66.8	OD	Female	Asian	Glaucoma suspect	0.75	7.66	absent	0	absent	2.21	1.96	1.73	1.88
34	57.4	OD	Female	Caucasian	Normal	1	7.27	absent	4	absent	2.02	1.38	1.64	1.78
35	48.3	OS	Female	Caucasian	Glaucoma suspect	-0.5	7.41	absent	13	present	1.66	1.68	1.31	1.45
36	57	OD	Male	Asian	Glaucoma suspect	0.88	8.09	absent	90	absent	2.45	2.08	1.54	1.68
37	49.9	OS	Male	Asian	Glaucoma suspect	0	7.45	absent	9	absent	2.89	2.22	2.2	2.34
38	48	OD	Male	Caucasian	Glaucoma suspect	0.5	7.75	present	17	absent	3.09	2.93	2.3	2.43
39	45.6	OD	Female	Caucasian	Glaucoma suspect	-0.63	7.52	absent	5	absent	2.85	2.15	1.97	2.1
40	58.8	OD	Male	Caucasian	Normal	-0.5	7.51	absent	8	absent	2.18	1.62	1.87	2
41	45.8	OD	Female	Asian	Glaucoma suspect	-1.25	7.54	present	17	present	2.51	1.66	1.88	2.01
42	61.4	OS	Male	Caucasian	Glaucoma suspect	0.63	7.63	absent	19	absent	2.28	1.93	1.78	1.91
43	49.7	OS	Male	Caucasian	Glaucoma suspect	-0.75	7.89	present	12	absent	3.47	2.82	2.62	2.74
44	63.8	OS	Female	Asian	Glaucoma suspect	1.5	7.35	absent	48	absent	2	1.76	1.54	1.66
45	68.7	OS	Male	Caucasian	Glaucoma suspect	2.5	7.86	absent	22	absent	1.66	1.59	1.36	1.47
46	47.4	OD	Female	Caucasian	Glaucoma suspect	-2.88	7.58	present	0	absent	2.86	2.4	1.87	1.98
47	61.2	OS	Female	Caucasian	Glaucoma suspect	-0.13	7.68	present	17	absent	2.01	1.67	1.73	1.83
48	41.8	OD	Male	Caucasian	Normal	0.63	7.71	present	19	absent	2.47	1.83	1.88	1.98
49	53.5	OS	Male	Caucasian	Glaucoma suspect	1	7.8	absent	18	absent	3.27	2.84	2.47	2.57

50	59.7	OS	Female	Asian	Glaucoma suspect	-0.63	7.26	absent	16	absent	3.32	2.58	2.52	2.61
51	52.3	OS	Male	Caucasian	Glaucoma suspect	1.75	7.74	present	82	absent	1.81	1.36	1.5	1.58
52	58.5	OD	Male	Caucasian	Glaucoma suspect	0.75	7.82	present	22	absent	2.32	1.54	1.48	1.56
53	56.9	OD	Female	Asian	Glaucoma suspect	2.38	7.72	absent	10	present	2.7	2.4	2.26	2.34
54	59.1	OD	Female	Caucasian	Glaucoma suspect	0.88	7.74	absent	10	absent	3.26	2.85	2.48	2.56
55	41.9	OD	Female	Asian	Normal	-1.75	7.68	absent	3	absent	3.73	2.95	2.46	2.54
56	60.3	OS	Female	Asian	Glaucoma suspect	0.75	7.73	absent	22	absent	2.61	2.13	1.84	1.92
57	59.4	OS	Male	Caucasian	Glaucoma suspect	1	7.72	present	29	absent	2.89	2.59	2.57	2.64
58	62.4	OS	Male	Caucasian	Normal	0.63	7.64	present	34	absent	2.9	2.6	2.31	2.38
59	58	OS	Male	Asian	Glaucoma	-0.38	7.75	present	3	present	3.86	3.36	2.95	3.02
60	55.3	OD	Female	Asian	Glaucoma suspect	0.25	7.37	absent	24	absent	2.8	2.31	2.27	2.33
61	46.1	OS	Male	Caucasian	Normal	-0.25	7.82	absent	0	absent	2.35	2.3	1.69	1.75
62	46	OD	Male	Caucasian	Glaucoma suspect	-0.5	7.77	absent	0	absent	3.12	2.68	2.2	2.26
63	42.3	OD	Male	Caucasian	Normal	-1.25	7.57	present	90	absent	2.13	1.76	1.71	1.76
64	61.5	OS	Female	Asian	Glaucoma suspect	2	7.83	absent	37	absent	2.01	1.82	1.67	1.72
65	55.7	OS	Female	Caucasian	Glaucoma suspect	-0.25	7.66	absent	21	absent	2.5	1.9	1.69	1.74
66	25	OD	Female	Caucasian	Glaucoma suspect	-1.25	7.73	absent	18	absent	2.07	1.81	1.53	1.58
67	67.4	OS	Female	Caucasian	Glaucoma suspect	1.63	7.89	absent	11	absent	2.14	1.72	1.43	1.48
68	61.7	OS	Male	Caucasian	Glaucoma	0.75	8.03	present	14	absent	2.31	2.12	1.73	1.78
69	70.4	OD	Male	Caucasian	Glaucoma suspect	1.38	7.64	present	15	absent	1.98	1.7	1.58	1.63
70	56.6	OD	Male	Caucasian	Glaucoma suspect	0	7.76	present	17	absent	3.37	2.36	2.59	2.63
71	42.8	OD	Male	Asian	Normal	-0.75	7.32	absent	15	absent	2.67	2.12	2.01	2.05
72	64.8	OD	Male	Caucasian	Glaucoma suspect	-2.25	7.41	present	52	present	1.29	1.15	1	1.04
73	67.4	OD	Male	Caucasian	Glaucoma suspect	-0.25	7.66	absent	0	absent	2.49	2.1	1.91	1.95
74	63.1	OD	Female	Caucasian	Glaucoma suspect	-0.25	7.81	absent	14	absent	2.83	2.34	2	2.04
75	32.8	OS	Male	Caucasian	Glaucoma suspect	-2	7.6	absent	22	absent	1.93	1.84	1.35	1.39
76	45	OD	Male	Asian	Glaucoma suspect	0.38	7.81	absent	11	present	2.48	2.04	1.88	1.91
77	61.7	OD	Male	Asian	Normal	-0.75	7.83	absent	12	absent	2.88	2.49	2.12	2.15
78	41.2	OD	Male	Caucasian	Glaucoma suspect	-0.75	7.82	absent	0	absent	2.77	2.1	2.04	2.06
79	57.1	OD	Male	Asian	Glaucoma suspect	1.38	7.95	present	8	absent	2.17	1.89	1.72	1.74
80	57.1	OD	Male	Caucasian	Glaucoma suspect	0.13	7.69	absent	7	absent	1.89	1.61	1.4	1.42
81	44.3	OS	Male	Caucasian	Glaucoma suspect	0	7.8	present	22	absent	2.88	2.47	2.26	2.28
82	54.3	OS	Male	Caucasian	Glaucoma suspect	-5	7.23	absent	14	absent	1.92	1.84	1.4	1.42
83	68.3	OD	Female	Caucasian	Glaucoma suspect	2.75	7.65	absent	0	absent	2.29	2.1	1.86	1.88
84	63.3	OD	Female	Caucasian	Glaucoma suspect	-2.13	7.87	absent	1	present	2.86	2.26	2.09	2.1
85	56.2	OD	Female	Caucasian	Glaucoma suspect	-1.13	7.79	present	4	absent	2.77	2.42	2.03	2.04
86	48.3	OS	Female	Caucasian	Glaucoma	0.63	7.79	absent	9	absent	1.94	1.21	1.43	1.43
87	66.5	OS	Female	Caucasian	Glaucoma suspect	-0.25	7.5	absent	0	absent	1.87	1.8	1.74	1.74
88	46.1	OD	Female	Caucasian	Glaucoma suspect	-0.13	7.6	absent	5	absent	2.97	2.42	2.2	2.2
89	53.2	OD	Male	Caucasian	Glaucoma suspect	1	7.7	present	1	absent	2.41	2.53	2.3	2.3
90	56.8	OD	Male	Caucasian	Glaucoma suspect	0.63	8.14	absent	70	absent	2.83	2.37	2.09	2.08
91	77.5	OD	Female	Caucasian	Glaucoma suspect	0.5	7.68	absent	55	absent	1.99	1.87	1.51	1.5
92	60.5	OS	Male	Caucasian	Glaucoma suspect	-0.25	7.76	present	16	absent	2.54	2.01	2.04	2.03
93	49.5	OS	Male	Caucasian	Glaucoma suspect	-0.25	7.64	present	58	absent	2.89	2.51	2.26	2.24
94	47.6	OD	Male	Asian	Glaucoma suspect	0.38	7.87	absent	9	absent	3.28	2.78	2.51	2.49
95	55.5	OD	Female	Asian	Glaucoma suspect	-0.25	7.72	absent	21	absent	2.3	1.88	1.64	1.62
96	72.8	OS	Male	Caucasian	Glaucoma suspect	1	7.64	absent	9	present	2.7	2.38	2.45	2.43
97	64.2	OD	Male	Caucasian	Glaucoma suspect	1.25	7.87	present	15	absent	2.34	2.19	2.04	2.02
98	49.6	OD	Male	Asian	Glaucoma suspect	-2.25	7.79	present	30	absent	2.27	2.02	1.83	1.81
99	45.3	OS	Female	Caucasian	Glaucoma suspect	-0.88	7.55	absent	7	absent	2.93	2.57	2.37	2.35

100	54.3	OD	Male	Caucasian	Glaucoma	1.63	8	absent	27	absent	2.73	2.09	2.05	2.02
101	40.4	OD	Male	Asian	Glaucoma	-1.25	8.04	present	13	absent	2.4	2.18	1.81	1.78
102	40.3	OS	Female	Caucasian	Glaucoma suspect	-0.88	8	absent	21	absent	3.44	2.86	2.35	2.32
103	52.6	OS	Female	Asian	Glaucoma suspect	0.75	7.51	present	17	absent	1.84	1.65	1.65	1.61
104	51.7	OS	Male	Caucasian	Glaucoma suspect	-4.38	7.55	absent	30	absent	2.87	2.39	1.88	1.84
105	50.5	OD	Male	Asian	Glaucoma suspect	-1	7.73	absent	22	present	3.05	2.45	2.65	2.61
106	64.5	OD	Male	Caucasian	Glaucoma suspect	-2.38	7.91	absent	8	absent	3.19	2.73	2.15	2.11
107	32.3	OS	Male	Caucasian	Glaucoma suspect	-0.88	7.91	absent	23	absent	2.8	2.4	2.01	1.96
108	66.8	OS	Female	Caucasian	Glaucoma suspect	0.75	7.76	absent	14	absent	1.94	1.57	1.49	1.44
109	49.6	OS	Female	Caucasian	Glaucoma suspect	-0.88	8	absent	78	absent	3.39	2.83	2.35	2.3
110	35.6	OD	Male	Caucasian	Glaucoma	-1.38	7.63	absent	5	present	3.01	2.82	2.51	2.45
111	38.2	OS	Male	Asian	Glaucoma suspect	-4.13	7.65	present	35	present	1.71	1.61	2.74	2.68
112	29.1	OD	Female	Asian	Glaucoma suspect	0	7.35	absent	24	absent	2.99	2.39	2.47	2.41
113	67.8	OS	Female	Caucasian	Glaucoma suspect	1.13	7.6	present	38	absent	2.21	1.84	1.87	1.81
114	47.1	OD	Male	Caucasian	Glaucoma suspect	-1.63	8.39	present	31	absent	1.9	1.65	1.46	1.4
115	50.7	OS	Female	Asian	Glaucoma suspect	-1.5	7.69	absent	8	absent	3	2.56	2.34	2.28
116	56.3	OS	Female	Asian	Glaucoma suspect	0.5	7.17	absent	0	absent	2.33	2.42	2.11	2.05
117	21.8	OD	Male	Asian	Glaucoma suspect	-4.5	7.91	absent	18	absent	2.35	1.94	1.44	1.36
118	51	OD	Female	Caucasian	Glaucoma suspect	-1.75	7.54	present	32	present	2.4	1.87	1.96	1.88
119	55.1	OD	Female	Asian	Glaucoma	-2.5	7.76	present	16	present	1.9	1.79	1.53	1.45
120	51.6	OD	Male	Caucasian	Glaucoma suspect	-2.25	7.78	absent	25	absent	4.36	4.09	3.4	3.32
121	48.7	OD	Female	Asian	Glaucoma suspect	0.38	8	absent	7	absent	3.53	3.04	2.78	2.69
122	52.5	OS	Female	Asian	Glaucoma suspect	-0.63	7.69	absent	40	absent	2.5	1.95	1.98	1.89
123	60.9	OS	Female	Asian	Glaucoma suspect	1.5	8.18	absent	15	absent	2.72	2.33	2.08	1.99
124	43	OS	Male	Caucasian	Normal	-0.13	7.99	present	31	absent	2.66	2.33	2.06	1.97
125	41.7	OD	Female	Asian	Glaucoma suspect	-3.25	7.63	absent	7	absent	3.1	2.1	2.14	2.05
126	57.5	OD	Male	Asian	Glaucoma suspect	-1	7.51	present	39	present	1.74	1.43	1.9	1.8
127	52.9	OD	Female	Caucasian	Glaucoma suspect	-4.13	7.39	absent	32	absent	2.16	1.6	1.54	1.44
128	38.9	OD	Female	Asian	Glaucoma suspect	-0.75	7.86	absent	30	absent	2.97	2.3	2.13	2.03
129	38.7	OS	Female	Asian	Glaucoma suspect	-0.25	7.68	absent	9	absent	2.23	2.04	1.91	1.8
130	46.8	OS	Female	Caucasian	Glaucoma suspect	-0.63	8.13	absent	6	absent	3.83	3.07	2.81	2.7
131	27.9	OD	Female	Asian	Normal	-0.63	7.7	absent	18	absent	3.69	3.61	2.85	2.74
132	60.1	OD	Male	Caucasian	Glaucoma suspect	0.38	8.09	present	68	absent	2.81	2.49	2.37	2.26
133	60.8	OD	Female	Caucasian	Glaucoma suspect	2.13	8	absent	18	absent	2.87	2.66	2.47	2.36
134	55.1	OS	Male	Caucasian	Glaucoma suspect	-0.13	8.04	absent	14	absent	3.07	2.8	2.14	2.03
135	58.6	OS	Female	Caucasian	Glaucoma	0.88	7.76	present	8	absent	2.3	2.34	2.03	1.91
136	57.7	OS	Male	Caucasian	Glaucoma suspect	-1.5	7.87	present	13	absent	2.52	2.11	2.12	2
137	53	OS	Male	Caucasian	Glaucoma suspect	0.13	8.34	absent	20	absent	3.11	2.49	2.38	2.26
138	66.8	OS	Male	Caucasian	Glaucoma suspect	-1.5	7.59	present	21	absent	2.19	1.79	1.55	1.43
139	30.2	OS	Female	Asian	Glaucoma suspect	-0.25	7.81	absent	10	absent	2.05	2.24	1.87	1.75
140	33.5	OS	Male	Caucasian	Glaucoma suspect	-1.75	7.71	absent	22	absent	2.51	1.81	1.9	1.77
141	66	OS	Male	Asian	Glaucoma suspect	1.5	7.77	present	42	absent	3.03	2.85	2.71	2.58
142	54	OD	Male	Asian	Normal	-2.88	7.34	absent	43	absent	2.84	2.83	2.34	2.2
143	30.3	OD	Male	Asian	Glaucoma suspect	-4	7.95	absent	24	absent	3.25	2.33	2.14	2
144	59.3	OS	Female	Caucasian	Glaucoma suspect	-1.25	7.74	absent	13	absent	2.61	2.19	2.07	1.91
145	65.4	OD	Male	Asian	Glaucoma suspect	-0.13	8.35	absent	13	absent	2.64	2.24	1.95	1.79
146	21.3	OS	Male	Caucasian	Glaucoma suspect	-1.75	8.17	absent	3	absent	4.29	3.8	3.19	3.03
147	47	OD	Female	Caucasian	Normal	-0.63	7.91	absent	4	absent	2.65	1.94	2.17	2
148	46.5	OD	Male	Caucasian	Glaucoma suspect	-1.38	8.04	absent	0	absent	3.86	2.96	2.79	2.62
149	44.8	OD	Female	Caucasian	Glaucoma suspect	-0.5	7.79	absent	28	absent	3.82	3.16	2.82	2.65

150	55.4	OS	Male	Caucasian	Glaucoma suspect	0.13	8.13	absent	26	absent	2.74	2.67	2.28	2.11
151	40.1	OD	Male	Asian	Glaucoma suspect	-0.25	8.24	absent	8	absent	3.55	2.38	2.68	2.51
152	49	OS	Male	Asian	Glaucoma suspect	0.38	7.96	absent	33	absent	3.14	2.9	2.45	2.28
153	55	OD	Female	Caucasian	Glaucoma suspect	1.75	8.21	absent	17	absent	3.16	2.19	2.32	2.14
154	71.7	OS	Female	Caucasian	Glaucoma suspect	-0.88	7.54	present	14	absent	2.48	2.44	2.27	2.09
155	38.7	OS	Male	Asian	Normal	0.13	8.19	absent	90	absent	3.61	3	2.86	2.67
156	62.3	OD	Female	Caucasian	Glaucoma suspect	-1.5	8.14	absent	18	present	2.74	2.18	2.05	1.85
157	57.6	OS	Male	Caucasian	Glaucoma suspect	-4.5	7.68	absent	1	absent	2.38	2.02	1.91	1.71
158	42.9	OS	Female	Caucasian	Glaucoma suspect	-3.75	7.52	absent	84	absent	2.99	2.54	2.25	2.04
159	52.4	OD	Male	Caucasian	Glaucoma suspect	-5.38	7.71	present	5	absent	3.44	2.45	2.16	1.95
160	50.7	OD	Male	Asian	Glaucoma suspect	-0.25	7.69	absent	82	absent	2.72	2.2	2.22	2
161	51.8	OS	Female	Caucasian	Glaucoma suspect	-0.38	8.13	absent	7	absent	3.45	2.76	2.72	2.49
162	57.2	OS	Female	Asian	Glaucoma suspect	-0.5	7.61	absent	28	present	2.18	2	1.92	1.69
163	61.4	OD	Female	Asian	Glaucoma	-2.63	7.83	present	9	present	2.69	2.34	2.19	1.96
164	38.4	OS	Male	Asian	Glaucoma suspect	-2.88	8	absent	28	absent	3.8	3.08	2.83	2.6
165	57.4	OD	Male	Caucasian	Glaucoma suspect	-0.13	7.92	absent	0	absent	2.25	1.15	1.98	1.74
166	45.3	OS	Male	Caucasian	Normal	-1.63	7.92	absent	3	absent	2.87	2.56	2.52	2.28
167	43.4	OS	Male	Asian	Glaucoma suspect	-0.13	8.26	present	17	absent	2.5	2.38	2.22	1.98
168	49.5	OS	Female	Caucasian	Glaucoma suspect	-3.38	7.85	absent	20	absent	3.3	2.91	2.58	2.34
169	75.9	OD	Male	Caucasian	Glaucoma	2	7.92	present	42	present	1.64	1.5	1.8	1.55
170	42.1	OD	Male	Caucasian	Glaucoma suspect	-0.25	8.02	present	16	absent	2.7	2.83	2.26	1.99
171	53.8	OD	Male	Asian	Glaucoma suspect	0.75	8.2	present	14	absent	3.46	2.73	2.74	2.47
172	59.3	OD	Female	Caucasian	Glaucoma suspect	1.63	8.14	absent	62	absent	2.49	2.2	2.05	1.77
173	53.3	OD	Female	Caucasian	Glaucoma suspect	-1.63	8.16	present	30	absent	3.54	3.05	2.53	2.24
174	67.6	OD	Male	Caucasian	Glaucoma	-2.13	7.82	present	21	absent	2.02	2.37	2.06	1.77
175	53.6	OS	Male	Asian	Glaucoma suspect	0.88	7.74	absent	8	absent	2.42	2.07	2.22	1.92
176	47.7	OS	Male	Caucasian	Glaucoma suspect	-0.38	8.22	absent	2	absent	4.46	3.24	3.16	2.85
177	66	OS	Female	Asian	Normal	0	7.7	absent	12	absent	2.59	2.07	2.55	2.22
178	64.9	OD	Female	Caucasian	Glaucoma suspect	2.88	7.71	present	16	absent	2.56	1.5	1.79	1.45
179	60.1	OD	Male	Caucasian	Glaucoma suspect	-3.75	8.07	absent	6	absent	3.23	2.89	2.48	2.12
180	44.8	OS	Female	Caucasian	Glaucoma suspect	-1.38	7.42	present	0	present	2.63	2.17	2.64	2.28
181	63.3	OS	Female	Caucasian	Glaucoma suspect	0	7.99	present	0	absent	2.91	2.59	2.53	2.16
182	53.2	OD	Female	Caucasian	Normal	0.25	7.68	absent	30	present	2.01	1.71	2.54	2.17
183	29.9	OS	Male	Caucasian	Normal	-6.38	7.49	present	28	absent	3.07	2.53	2.34	1.94
184	46.7	OS	Male	Asian	Glaucoma suspect	-2.25	7.78	present	97	absent	3.34	3.12	2.87	2.46
185	33.8	OD	Male	Asian	Glaucoma suspect	-5.5	7.75	absent	3	present	3.67	2.4	2.71	2.29
186	49.4	OS	Male	Caucasian	Glaucoma suspect	1.13	8.25	present	13	absent	2.88	2.81	2.78	2.35
187	60.5	OD	Male	Caucasian	Glaucoma suspect	2.63	8.33	present	72	absent	3.01	2.58	2.72	2.29
188	66.7	OS	Female	Asian	Glaucoma	-2.13	7.33	present	27	present	1.68	1.49	1.96	1.52
189	31.7	OS	Male	Asian	Glaucoma suspect	-1.25	7.55	present	5	present	2.33	1.9	2.3	1.85
190	36.6	OS	Female	Asian	Glaucoma suspect	-1.63	7.79	absent	13	present	2.12	1.66	2.21	1.76
191	55.5	OD	Female	Asian	Glaucoma suspect	-1.63	7.79	absent	17	present	2.27	1.88	2.22	1.77
192	44.9	OS	Male	Asian	Glaucoma suspect	0.25	7.71	present	41	present	1.53	1.2	2.3	1.84
193	32.5	OS	Male	Asian	Glaucoma suspect	-5.13	7.46	absent	0	absent	2.88	2.51	2.51	2.01
194	61	OD	Female	Asian	Glaucoma	0.75	7.84	present	97	present	2.82	2.56	2.82	2.31
195	26.1	OD	Male	Asian	Normal	-3.88	8.08	absent	18	absent	3.82	2.65	2.67	2.15
196	64	OS	Female	Caucasian	Glaucoma	-0.38	7.93	present	0	absent	2.59	2.19	2.7	2.16
197	57	OS	Female	Caucasian	Glaucoma suspect	-2.38	7.82	present	32	present	1.32	1.38	1.77	1.23
198	72.9	OS	Male	Caucasian	Glaucoma suspect	-1.5	7.6	absent	18	absent	2.49	1.74	1.96	1.42
199	63.2	OD	Male	Caucasian	Glaucoma suspect	-5	7.73	absent	9	present	2.48	2.28	2.14	1.59

200	48.2	OS	Female	Caucasian	Glaucoma suspect	-2.38	8.01	absent	26	absent	2.12	1.99	2.31	1.75
201	60.1	OD	Male	Caucasian	Glaucoma suspect	0.25	8.02	present	16	present	2.64	2.26	2.6	1.98
202	46.2	OS	Male	Asian	Glaucoma suspect	-2.25	7.99	present	11	absent	3.1	3.11	2.91	2.28
203	22.6	OD	Female	Asian	Glaucoma	-4.25	8.02	absent	11	present	2.9	2.8	2.38	1.74
204	66.2	OD	Male	Caucasian	Glaucoma suspect	-3.88	7.8	present	4	absent	2.98	2.68	3.05	2.35
205	43	OS	Male	Asian	Glaucoma suspect	-1.88	8.16	present	3	absent	2.8	2.43	2.77	2.03
206	53.4	OD	Female	Asian	Glaucoma	-4.5	8.03	present	37	present	3.62	2.97	3.3	2.34
207	47.1	OD	Female	Caucasian	Glaucoma suspect	-4.5	7.7	present	3	present	2.3	1.95	2.54	1.57
208	53.2	OS	Female	Caucasian	Glaucoma suspect	-3.75	7.71	present	41	present	1.76	1.42	2.34	1.26
209	44.7	OD	Female	Asian	Glaucoma suspect	-5.5	7.79	present	0	present	3.03	2.49	3.24	2.09
